# Ventromedial Hypothalamus Activation Aggravates Hypertension Myocardial Remodeling Through the Sympathetic Nervous System

**DOI:** 10.3389/fcvm.2021.737135

**Published:** 2021-10-18

**Authors:** Yuyang Zhou, Zhihao Liu, Zihan Liu, Huixin Zhou, Xiao Xu, Zeyan Li, Hu Chen, Yuhong Wang, Zhen Zhou, Meng Wang, Yanqiu Lai, Liping Zhou, Xiaoya Zhou, Hong Jiang

**Affiliations:** ^1^Department of Cardiology, Renmin Hospital of Wuhan University, Wuhan, China; ^2^Cardiac Autonomic Nervous Research Center, Wuhan University, Wuhan, China; ^3^Department of Cardiology Cardiovascular Research Institute, Wuhan University, Wuhan, China; ^4^Hubei Key Laboratory of Cardiology, Wuhan, China

**Keywords:** ventromedial hypothalamus, sympathetic nervous system, hypertension, cardiac remodeling, HIF-1α, PPARα, CPT-1

## Abstract

**Background:** The ventromedial hypothalamus (VMH) is an important nuclei in responding to emotional stress, and emotional stress is a risk factor for cardiovascular diseases. However, the role of the VMH in cardiovascular diseases remains unknown. This study aimed to investigate the effects and underlying mechanisms of VMH activation on hypertension related cardiac remodeling in two-kidney-one-clip (2K1C) hypertension (HTN) rats.

**Methods:** Eighteen male Sprague-Dawley rats were injected with AAV-hSyn-hM3D(Gq) into the VMH at 0 weeks and then randomly divided into three groups: (1) sham group (sham 2K1C + saline i.p. injection); (2) HTN group (2K1C + saline i.p. injection); (3) HTN+VMH activation group (2K1C + clozapine-N-oxide i.p. injection). One week later, rats were subjected to a sham or 2K1C operation, and 2 weeks later rats were injected with clozapine-N-oxide or saline for 2 weeks.

**Results:** In the HTN+VMH activation group, FosB expression was significantly increased in VMH sections compared with those of the other two groups. Compared to the HTN group, the HTN+VMH activation group showed significant: (1) increases in systolic blood pressure (SBP); (2) exacerbation of cardiac remodeling; and (3) increases in serum norepinephrine levels and sympathetic indices of heart rate variability. Additionally, myocardial RNA-sequencing analysis showed that VMH activation might regulate the HIF-1 and PPAR signal pathway and fatty acid metabolism. qPCR results confirmed that the relative mRNA expression of HIF-1α was increased and the PPARα and CPT-1 mRNA expression were decreased in the HTN+VMH activation group compared to the HTN group.

**Conclusions:** VMH activation could increase SBP and aggravate cardiac remodeling possibly by sympathetic nerve activation and the HIF-1α/PPARα/CPT-1 signaling pathway might be the underlying mechanism.

## Introduction

The ventromedial hypothalamus (VMH) is an important emotion-related nuclei that responds to emotional stress and is involved in the regulation of emotional reactions. The VMH can be activated by emotional stress derived from environmental threats (1). Moreover, the activation of VMH nuclei by pharmacogenetics or optogenetic techniques could cause a variety of emotional reactions, such as anxiety, fear and anger (2–4). VMH activation plays an important role in emotional stress, which is a risk factor and prognostic factor for cardiovascular diseases (CVDs) (5). Clinical studies have found that patients who experience prolonged emotional stress were more likely to develop CVDs (6, 7). Moreover, emotional stress could increase the recurrence and death risk of adverse cardiovascular events (8, 9). However, the role of VMH nuclei in CVDs is currently unclear.

The VMH has been demonstrated to modulate the sympathetic nervous system activity in the periphery. Lesions in the VMH could reduce sympathetic outflow (10). In contrast, VMH activation by electronic stimulation or drug injection could increase the sympathetic nervous system activity (11, 12). It is well-known that excessive sympathetic nervous system (SNS) activation is involved in the onset and development of cardiovascular disease (13). Overactivated sympathetic nerves could induce increased blood pressure, cardiac remodeling and impaired cardiac systolic or diastolic function (13–17), while inhibition of sympathetic activity improves cardiac remodeling and cardiac function (18, 19). Therefore, we speculated that the SNS is the key to the link between VMH activation and CVDs.

In the present study, using hM3D(Gq) (excitatory) Designer Receptors Exclusively Activated Designer Drugs (DREADDs) to specifically activate the VMH, we aimed to investigate whether the activation of the VMH could exacerbate cardiac remodeling and increase systolic blood pressure in hypertension (HTN) rats via increased the sympathetic nervous system activity, and to investigate the potential molecular mechanisms.

## Method

### Animals

All experimental protocols were approved by the Animal Care and Use Committees of Renmin Hospital of Wuhan University. Male Sprague-Dawley rats (180–230 g) were housed at a constant temperature (25 ± 2°C) with 12 h light and dark cycles and fed standard rat food.

### Experimental Design

**Figure 1:**
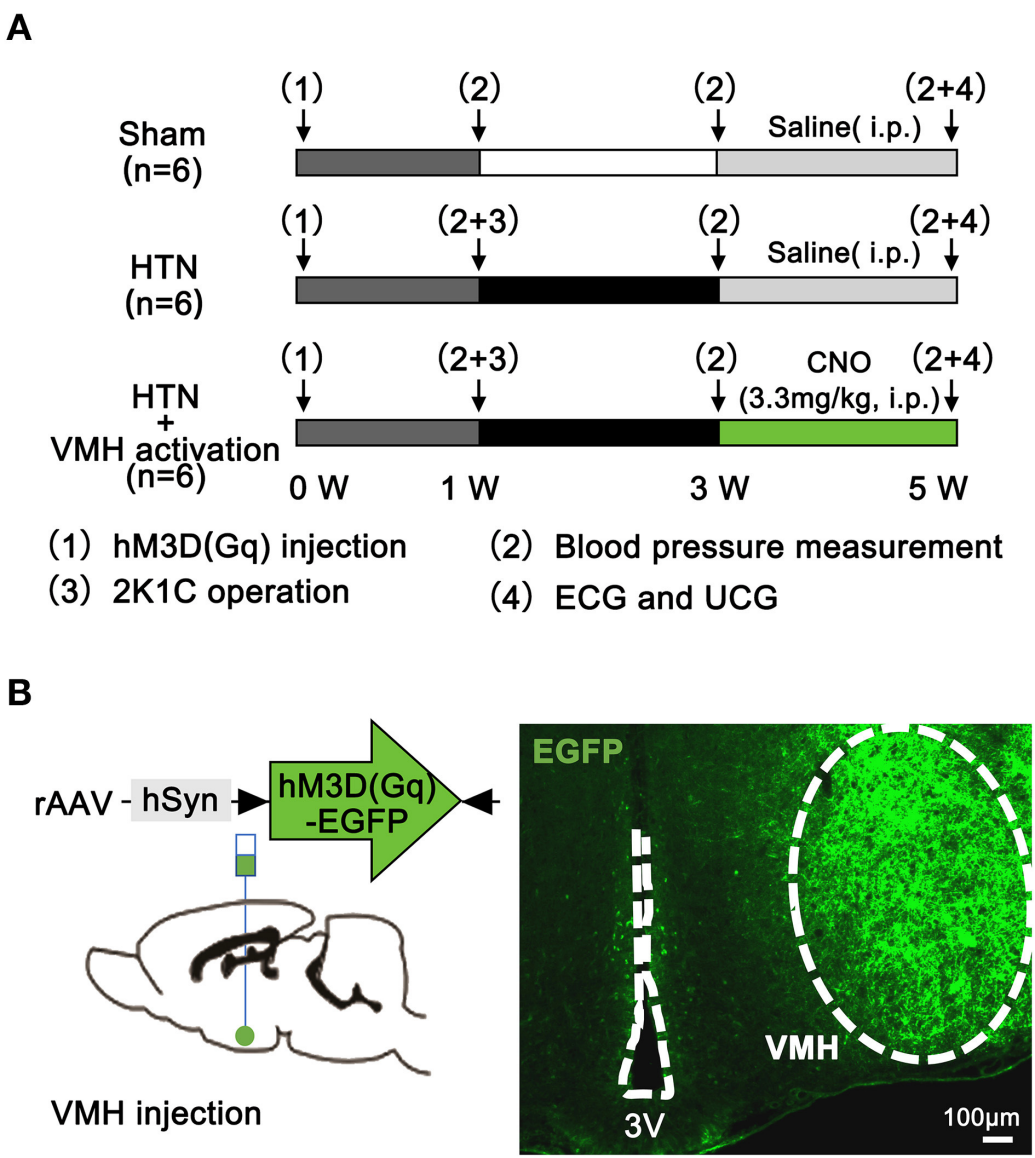
Experimental protocol and pharmacogenetics method. **(A)** Experimental protocol (*n* = 6 per group); **(B)** Schematic diagram and representative image showing virus injection and hM3D(Gq)-EGFP expression in the VMH at 3 weeks. The scale bar represents 100 μm. VMH, ventromedial hypothalamus; ECG, electrocardiogram; UCG, ultrasonic cardiogram; CNO, clozapine-N-oxide; 3V, the third ventricle.

All rats received rAAV-hSyn-hM3D(Gq)-EGFP-WPRE-pA2/2 virus injection at 0 weeks, and then rats were randomly divided into following 3 groups (Figure 1A). (1) Sham group: One week after the virus injection, rats were subjected to a sham two-kidney one-clip (2K1C) operation. Two weeks after the sham operation, saline was given intraperitoneally (i.p.) for 2 weeks (2) HTN group: One week after the virus injection, rats were subjected to a 2K1C operation. Two weeks after the 2K1C operation, saline was injected (i.p.) for 2 weeks (3) HTN+VMH activation group: One week after the virus injection, rats were subjected to a 2K1C operation. Two weeks after the 2K1C operation, clozapine-N-oxide (CNO, 3.3 mg/kg, i.p.) was injected for 2 weeks. At 1 week, blood pressure was measured in all rats before the sham or 2K1C operation. Then, 2 weeks later, the blood pressure measurement was performed in all three groups. After saline or CNO injection for 2 weeks, the rats were subjected to blood pressure, ultrasonic cardiogram (UCG), and surface electrocardiogram (ECG) measurements before they were sacrificed. Then, blood samples, hearts and brain tissues were collected.

### DREADDs Stereotaxic Injection

Rats were anesthetized with pentobarbital (2.5%) and placed in a stereotaxic instrument (RWD Life Science, Shenzhen, China). A midline skin incision was made on the head to expose the surface of the skull. 100 nl of rAAV-hSyn-hM3D(Gq)-EGFP-WPRE-pA2/2 virus (Brain VTA, Wuhan, China) was injected into the bilateral VMH (20) (coordinates: anteroposterior −2.6 mm, mediolateral ±0.6 mm, dorsoventral −9.2 mm) at an infusion rate of 10 nL/min using a 10-μl Hamilton nanoinjector (Figure 1B). The injector was left in place for 10 min before withdrawal. Rats were allowed to recover for 1 w before the 2K1C operation.

### Hypertension Model

Rats were anesthetized by i.p. injection of 2.5% pentobarbital. A silver clip (internal diameter 0.25 mm) was placed around the left renal artery. In the sham-operated group, animals underwent a similar surgical procedure without clip placement. After 2 weeks, blood pressure was measured. In the HTN and the HTN+VMH activation group, rats with systolic blood pressure (SBP) ≥150 mmHg were selected.

### Measurement of Blood Pressure

At 1, 3, and 5 w after VMH injection, rats in each group were placed in a warm incubator at 40°C for 15 min. Then, the tail artery pressure of each rat was measured with a caudal artery cuff (BP-2010A, Softron Beijing Biotechnology Co., Ltd., Beijing, China) in a calm and quiet state. Blood pressure was continuously recorded three times to calculate the average.

### Surface Electrocardiogram Recording

At 5 w, surface electrocardiogram (lead II) was then performed of each animal for 10 min (min) under anesthesia (2.5% pentobarbital sodium, i.p.). Heart rate variation (HRV) was analyzed from 5-min segments of ECG data with the HRV Module in LabChart software (ADInstruments, Castle Hill, NSW, Australia). The following power spectral variables were documented: the high frequency (HF, 0.75–2.5 Hz), the low frequency (LF, 0.20–0.75 Hz), and the LF/HF ratio.

### Blood Sample Collection and Serum Noradrenaline Detection

After the completion of the ECG, 1 ml of arteria blood was collected and centrifuged at 3000 rpm for 15 min, then stored at −80°C. The norepinephrine (NE) concentration was measured with ELISA kit (CEA907Ge, Cloud-Clone, USA) following the instructions.

### Histological and Immunofluorescence Analysis

The hearts and brains were rapidly removed, rinsed in ice cold 0.9% saline solution, blotted and fixed in phosphate-buffered 10% paraformaldehyde for histological study. Ventricles were isolated and cut into 2 fragments by a mid-ventricular coronal section. Each block was serially cut in the same direction into 4-μm thick sections. Hematoxylin and eosin stain (Servicebio, Wuhan, China) was used to determined myocytes cross area. Masson (Servicebio, Wuhan, China) stain was used for cardiac fibrosis analysis. TUNEL (Servicebio, Wuhan, China) was used to detect apoptosis of myocardial cells. Brains were cut into 5-μm sections for immunofluorescence analysis. The expression of Fos B (Abcam, USA) in VMH nuclei was used to evaluate neuronal activity. All analyses were quantified by Image Pro Plus (Media Cybernetics, Inc., Rockville, MD).

### Cardiac Transcriptomics and Analysis

To determine the cardiac genes and pathways influenced by VMH activation, the cardiac transcriptome of rats in the HTN group and HTN+VMH activation group was assessed. Left ventricular tissues were collected after rats were sacrificed and stored at −80°C before use. Total RNA was extracted using TRIzol reagent and purified with the RNeasy Mini Kit according to the instructions to enrich mRNA. Then, mRNA was reverse transcribed into cDNA. The BGSEQ-500 platform (BGI Inc., Wuhan, China) was used for gene sequencing to obtain raw reads. Clean reads were obtained from raw reads and then mapped to the rat reference genome (Rattus_norvegicus, UCSC, rn6). The expression values were calculated as fragments per kilo base per million mapped reads (FPKM) (RSEM software, default, v1.2.8). R software was used for hierarchical clustering analysis, Gene Ontology (GO) enrichment analysis and Kyoto Encyclopedia of Genes and Genomes (KEGG) pathway analysis of differentially expressed genes (DEGs) between different groups. *P*-value <0.05 was used as the threshold to select significant enrichment of the gene sets.

### qRT-PCR

Total RNA was extracted from LV tissue using TRIzol reagent (Invitrogen™, Thermo) according to the manual. RNA concentration was measured using a NanoDrop2000 (Thermo). Then, cDNA was synthesized by using a RevertAid First Strand cDNA Synthesis Kit (Thermo). Quantitative real-time PCR was performed to quantify the relative mRNA expression of hypoxia-inducible factor-1α (HIF-1α, forward primer: 5′-ATGCCAGATCACAGCACA-3′; reverse primer:5′-GGACAAACTCCCTCACCA-3′), peroxisome proliferator-activated receptor α (PPARα, forward primer:5′-TGTGCATGGCTGAGAAGA-3′; reverse primer:5′-CAGTGGAAGAATCGGACCT-3′), carnitine palmitoyl transferase 1 (CPT-1, forward primer:5′-TGACCCAAAGCAGTACCC-3′; reverse primer: 5′-CAGGACCAAAGCCACCT-3′), atrial natriuretic peptide (ANP, forward primer: 5′-CGGTACCGAAGATAACAGCCA-3′; reverse primer: 5′-TCACCACCTCTCAGTGGCAA-3′) and brain natriuretic peptide (BNP, forward primer: 5′-TTTCCTTAATCTGTCGCCGCT-3′; reverse primer: 5′-TGCATCGTGGATTGTTCTGGA-3′). Actin was used for normalization (forward primer:5′-CCTGTATGCCTCTGGTCGT-3′; reverse primer: 5′-CTGTAGCCACGCTCGG-3′).

### Statistics

SPSS 23.0 statistical software (SPSS Inc., Chicago, IL, USA) was applied for data analysis. The experimental results were expressed as the mean ± standard error of mean (SEM). Two-way ANOVA was used to analysis SBP. One-way ANOVA was used to analysis the rest of the data. Comparisons between any two means were performed by Tukey method. *p* < 0.05 represented that the difference was statistically significant.

## Results

### VMH Nuclei Was Activated by DREADDs

**Figure 2:**
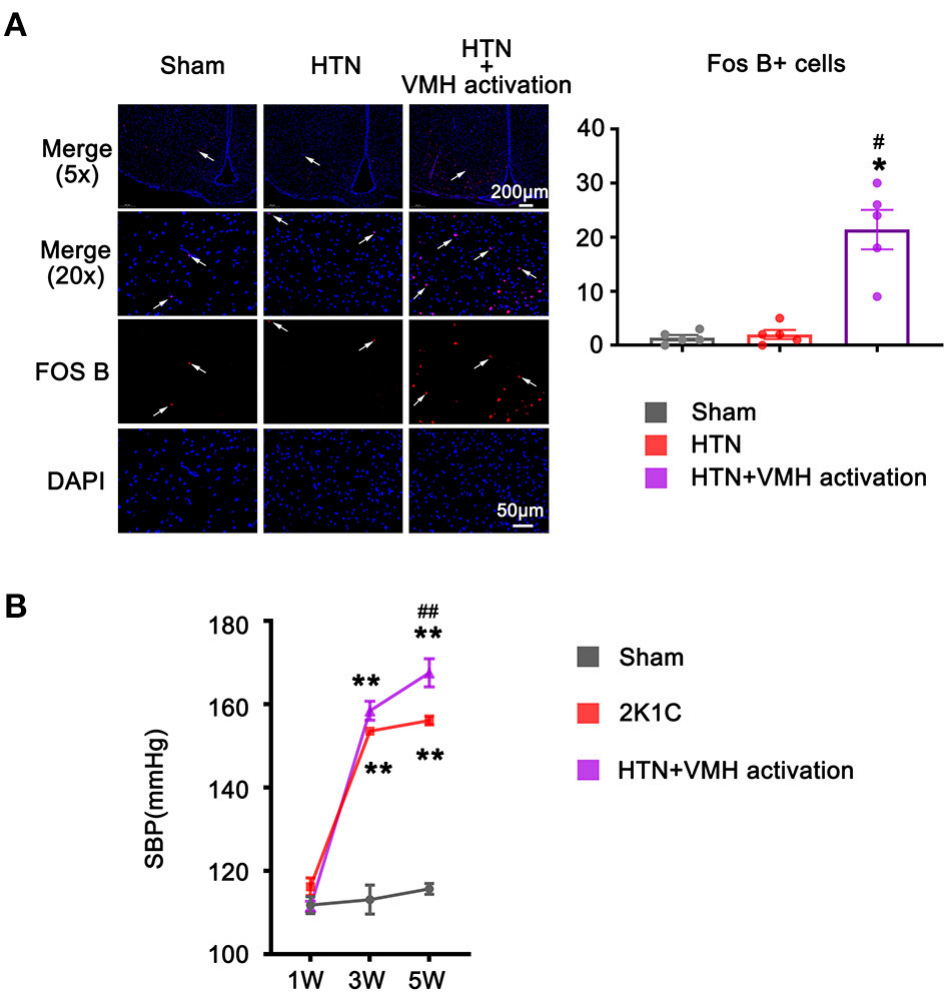
The increase of FOS B expression and systolic blood pressure after VMH activation. **(A)** Representative images (5x and 20x) and quantification of Fos B expression in the VMH. The scale bar represents 200 μm and 50 μm. Values are presented as mean ± SEM (*n* = 5 per group); **(B)** SBP was measured at 1W, 3W, and 5W. Values are presented as mean ± SEM (*n* = 6 per group). VMH, ventromedial hypothalamus; SBP, systolic blood pressure; *p < 0.05, **p < 0.01; vs. sham group; #*p* < 0.05, ##*p* < 0.01 vs. HTN group.

FosB expression, a marker of chronic neuronal activation, was significantly increased in VMH sections in the HTN+VMH activation group compared with sham group or HTN group, indicating that VMH neurons were successfully activated by DREADDs (1.40 ± 0.51 vs. 2.00 ± 0.84 vs. 21.40 ± 3.66, sham vs. HTN vs. HTN+VMH activation, *p* < 0.05, Figure 2A).

### Activation of VMH Nuclei Increased Systolic Blood Pressure

The baseline SBP recorded at 1 w showed no effective difference among the three groups (112 ± 2 mmHg vs. 116 ± 2 mmHg vs. 111 ± 1 mmHg, sham vs. HTN vs. HTN +VMH activation, *p* > 0.05). HTN treatment significantly elevated SBP at 3 w in the HTN group and HTN+VMH activation group compared to the sham group (113 ± 3 mmHg vs. 154 ± 1 mmHg vs. 159 ± 2 mmHg, sham vs. HTN vs. HTN+VMH activation, *p* < 0.001). At 5 w, VMH activation effectively elevated SBP in the HTN+VMH activation group compared with the HTN group (156 ± 1 mmHg vs. 168 ± 3 mmHg, HTN vs. HTN+VMH activation, *p* < 0.001, Figure 2B).

### Activation of VMH Nuclei Aggravated Cardiac Remodeling

**Figure 3:**
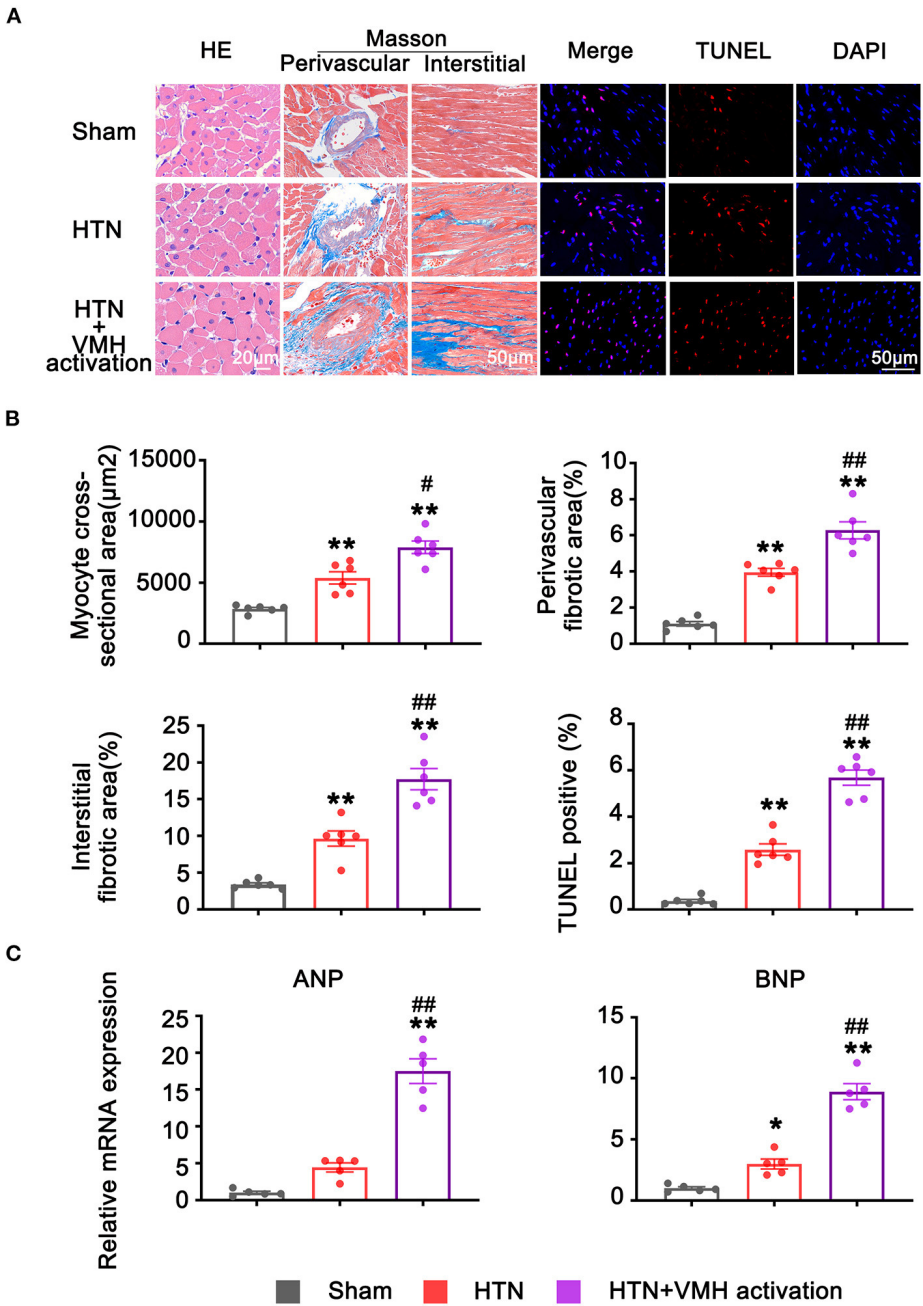
VMH activation aggravated cardiac remodeling. **(A)** Representative histological staining images (the scale bar represents 20 μm), Masson staining images (the scale bar represents 50 μm), and TUNEL staining images (the scale bar represents 50 μm) of left ventricle. **(B)** Quantification of myocytes cross-sectional areas, peripheral fibrosis areas, interstitial fibrosis areas, and TUNEL positive cells. Values are presented as mean ± SEM (*n* = 6 per group). **(C)** The relative mRNA expression of ANP and BNP. Values are presented as mean ± SEM (*n* = 5 per group). ANP, atrial natriuretic peptide; BNP, brain natriuretic peptide. *p < 0.05, **p < 0.01; vs. sham group; #*p* < 0.05, ##*p* < 0.01 vs. HTN group.

The HE staining results revealed cardiomyocyte hypertrophy in the HTN and HTN+VMH activation group compared with the sham group. Additionally, the HTN+VMH activation group showed aggravated cardiomyocyte hypertrophy compared to HTN group, which was confirmed by quantification of individual cardiomyocyte cross-sectional areas (2858 ± 125 μm^2^ vs. 5397 ± 501 μm^2^ vs. 7893 ± 510 μm^2^, sham vs. HTN vs. HTN +VMH activation, *p* < 0.01, Figure 3).

Cardiac fibrosis was assessed by Masson staining. Compared with the sham group, rats in the HTN group developed both perivascular and interstitial cardiac fibrosis (perivascular fibrotic area, 1.10 ± 0.12% vs. 3.95 ± 0.22%, sham vs. HTN, *p* < 0.01; interstitial fibrotic area, 3.39 ± 0.23% vs. 9.64 ± 1.04%, sham vs. HTN, *p* < 0.01). Additionally, compared with the HTN group, the HTN+VMH activation group showed further increases in cardiac fibrosis (perivascular fibrotic area, 3.95 ± 0.22% vs. 6.28 ± 0.47%, HTN vs. HTN+VMH activation, *p* < 0.05; interstitial fibrotic area, 9.64 ± 1.04% vs. 17.73 ± 1.46%, HTN vs. HTN+VMH activation, *p* < 0.01, Figure 3).

Myocyte apoptosis was assessed by TUNEL staining. The percentage of TUNEL positive cardiomyocytes per area of myocardium was statistically higher in the HTN+VMH activation group than in the HTN group (2.59 ± 0.25% vs. 5.69 ± 0.33%, HTN vs. HTN+VMH activation, *p* < 0.001, Figure 3).

To access the effect of VMH activation on hypertension-induced cardiac remodeling, we investigate the mRNA expression of ANP and BNP. The results suggested that in comparation with sham group, the relative mRNA expression of BNP was significantly increased in HTN group (*p* < 0.05). While, there were no significantly changes in ANP between the sham group and the HTN group (*p* > 0.05, Figure 3C). Additionally, VMH activation could significantly increase the relative expression of ANP and BNP mRNA compared with HTN group (ANP, *p* < 0.01; BNP, *p* < 0.01, Figure 3C).

### Activation of VMH Nuclei Increased Sympathetic Nervous System Activity

**Figure 4:**
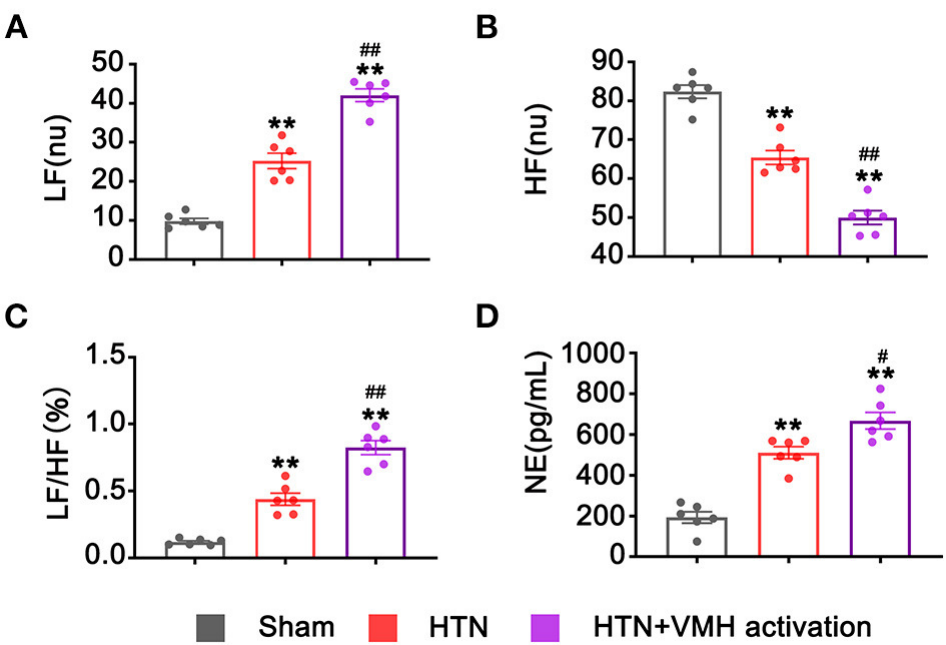
VMH activation increased sympathetic nervous system activity. **(A–C)** Spectral analysis of heart rate variability: LF, HF, and the ratio of LF/HF; **(D)** NE levels in serum. Values are presented as mean ± SEM (*n* = 6 per group). LF, low frequency (0.20–0.75 Hz); HF, high frequency (0.75–2.5 Hz); LF/HF, the LF/HF ratio; NE, norepinephrine. **p < 0.01; vs. sham group; ^#^*p* < 0.05, ^*##*^*p* < 0.01 vs. HTN group.

Heart rate variation and circulatory NE levels were used to evaluate the activity of the sympathetic nervous system. VMH activation significantly increased LF and the LF/HF ratio and markedly reduced HF compared with the HTN group (LF, 25.24 ± 1.98 nu vs. 42.06 ± 1.61 nu, HTN vs. HTN+VMH activation, *p* < 0.001; HF, 65.45 ± 1.79 nu vs. 49.99 ± 1.78 nu, HTN vs. HTN+VMH activation, *p* < 0.001, LF/HF, 0.44 ± 0.05% vs. 0.82 ± 0.05%, HTN vs. HTN+VMH activation, *p* < 0.001, Figures 4A–C). Similarly, the NE concentration in serum was significantly higher in the HTN+VMH activation group than the HTN group (511.03 ± 29.40 pg/mL vs. 667.82 ± 40.57 pg/mL, HTN vs. HTN+VMH activation, *p* < 0.05, Figure 4D).

### Myocardial Transcriptomic Changes Resulting From VMH Activation

**Figure 5:**
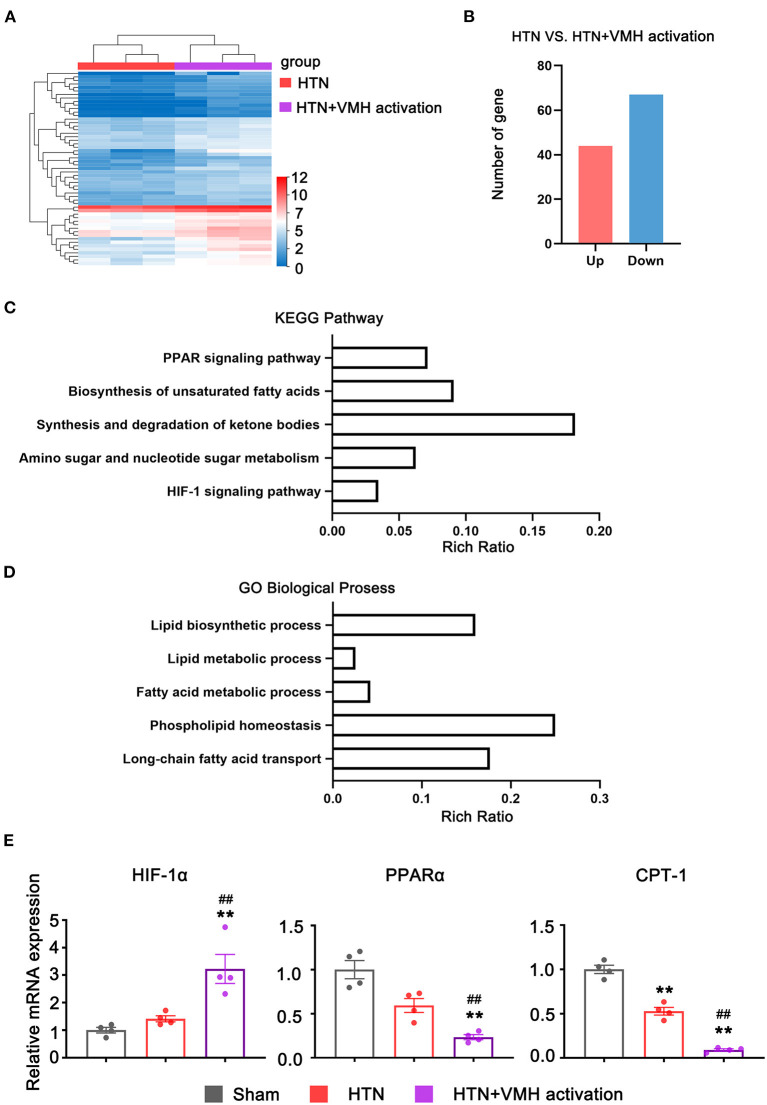
The RNA-seq and qPCR analysis results. **(A)** Heatmap of expression genes between HTN and HTN+VMH activation group (*n* = 3 per group); **(B)** Number of up-regulated and down-regulated genes between HTN and HTN+VMH activation group (*n* = 3 per group); **(C)** KEGG pathway enrichment analysis between HTN and HTN+VMH activation group; **(D)** GO biological process analysis between HTN and HTN+VMH activation group; **(E)** The relative mRNA expression of HIF-1α, PPARα, and CPT-1 in sham group, HTN group, and HTN+VMH activation group. Values are presented as mean ± SEM (*n* = 4 per group). **p < 0.01; vs. sham group; ##*p* < 0.01 vs. HTN group.

To investigate the effects of VMH activation on mRNA expression in the heart, RNA sequencing was used in the HTN group and HTN+VMH activation group. Figure 5A demonstrated the differential gene expression heatmap between the HTN group and the HTN+VMH activation group (*n* = 3). The data showed that 41 genes were upregulated and 55 genes were downregulated (Figure 5B). KEGG pathway enrichment analysis and GO biological process analysis (the top five results with the highest *P*-value were selected) revealed significantly changes in PPAR signaling pathway, HIF-1 signaling pathway and fatty acid metabolism process (*p* < 0.05, Figures 5C,D). We further analyzed the related genes in these signaling pathways by qPCR and showed that VMH activation significantly increased the expression of HIF-1α mRNA, and statistically decreased the expression of PPARα and CPT-1 mRNA (*p* < 0.05, Figure 5E).

## Discussion

### Major Findings

**Figure 6:**
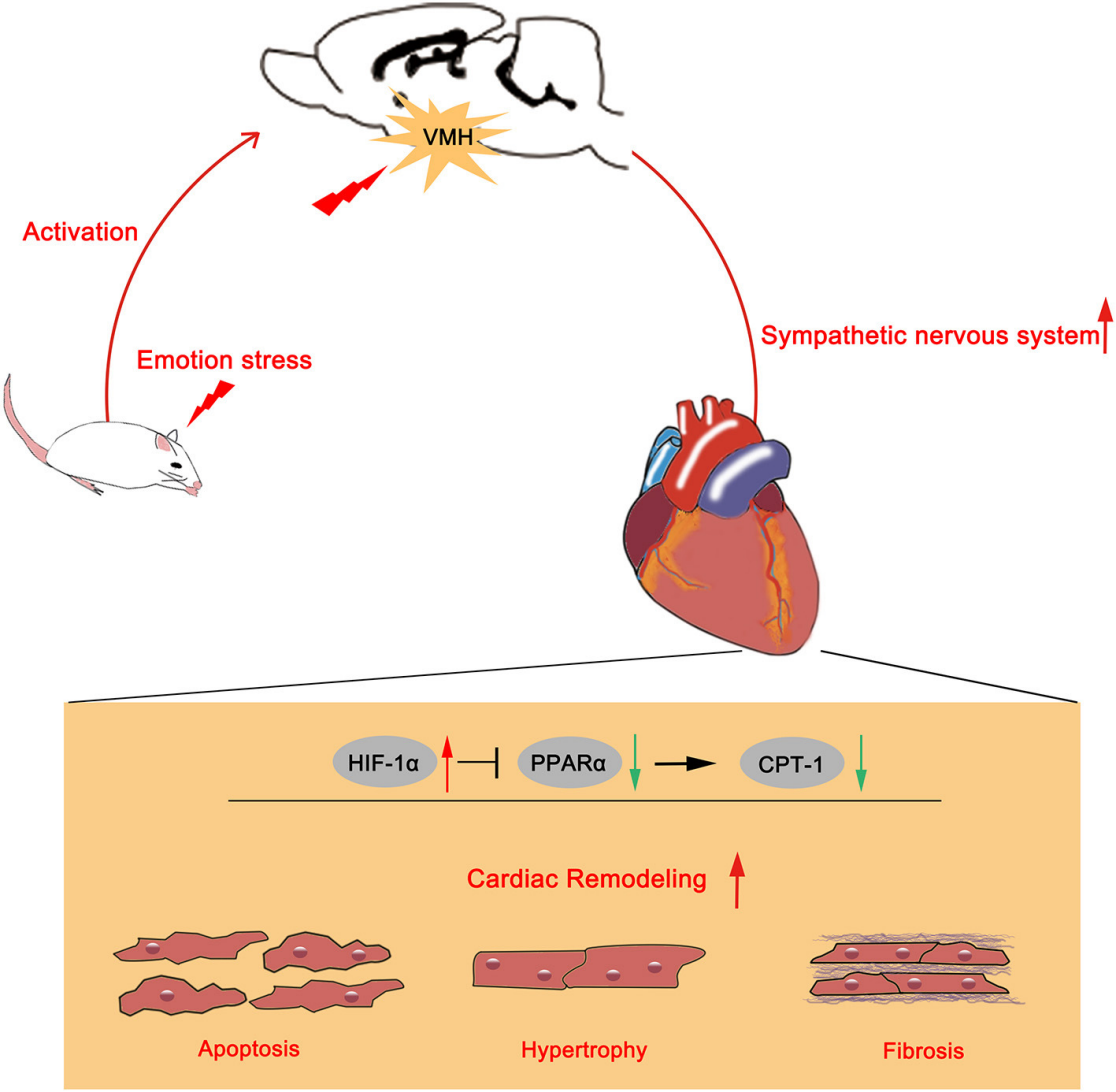
Schematic summary of the effects of VMH activation in HTN hypertension model. Emotional stress caused VMH activation could increase sympathetic nervous system activity. Upregulated sympathetic activity might increase HIF-1α expression and reduced PPARα and CPT-1 expression, then aggravated cardiac remodeling.

The present study provided evidence that VMH activation could increase systolic blood pressure and hypertension-related cardiac remodeling by activating sympathetic nervous system. However, the HIF-1α/PPARα/CPT-1 signaling pathway might be the potential underlying mechanism (Figure 6).

### VMH Activation Regulated Cardiovascular Function and Aggravated Cardiac Remodeling via the Sympathetic Nervous System

As one subnuclei of hypothalamus, VMH has been demonstrated to participate in emotional stress induced behavioral regulation (1, 21). The VMH has also been recognized to play an important role in modulating SNS outflow. Electrical stimulation or pharmacological (leptin, glucose, etc.) treatment of the VMH activated the sympathetic nervous system and increased serum norepinephrine levels (12, 22, 23). Rats with VMH lesions demonstrated downgraded sympathetic activity (10). As one of the vital connections between central brain nuclei and peripheral organs, the SNS could mediate the signal from the VMH to the cardiovascular system. Lim et al. (24) demonstrated that VMH activation engaged the sympathoexcitatory, leading to obesity-related hypertension. Additionally, Takenaka et al. (25) reported that baclofen (GABA receptor agonist) injected into the VMH decreased sympathetic nerve activity, BP and heart rate, and the reductions were significantly larger in spontaneously hypertensive rats than in Wistar-Kyoto rats. Furthermore, the depressor effects were abolished by intravenous pretreatment with phentolamine (adrenergic receptor blocker). The above findings indicate that VMH could modulate cardiovascular function via SNS activity.

In accordance with previous studies, the present study found that in comparison with the HTN group, the HTN+VMH activation group showed significantly increases in systolic blood pressure. Moreover, VMH activation could also statistically increase serum NE levels, LF and the LF/HF ratio, which have been used as markers of sympathetic activity.

### Activation of the SNS Might Aggravate Cardiac Remodeling via the HIF-1α/PPARα/CPT-1 Pathway

To investigate the molecular pathways that caused the deterioration of myocardial remodeling after VMH activation, we used RNA-Seq to identify the changes in the transcriptome of the heart. Ultimately, we filtered out the PPAR signaling pathway, HIF-1 signaling pathway and fatty acid metabolism.

In the pathological process of cardiac remodeling, myocardial energy source converts fatty acids to glucose. HIF-1α has been considered involving in the conversion of pathological myocardial glucolipid metabolism and targeted PPARα to interfere with glucolipid metabolism (26). Studies have proven that myocyte hypertrophy could activate HIF-1α expression (27, 28), and the increase in HIF-1α expression in hypertrophic myocardium could cause a decrease in PPARα expression (29). While, PPARα could regulate the downstream effector CPT-1 to mediate the uptake and oxidation of myocardial fatty acid. Studies have reported that the reduction in PPARα expression would cause the downregulation of CPT-1 (30), and partial loss of CPT-1 would further aggravate LV systolic dysfunction and cardiac remodeling in pressure overload model mice (31). Moreover, it has been shown that the sympathetic nervous system might be involved in the regulation of the above signaling pathways. Studies demonstrated that isoprenaline and phenylephrine could increase HIF-1α expression (32, 33) and inhibit PPARα and CPT-1 expression (34, 35).

Consistent with previous research, in this research, we found that VMH activation could aggravate hypertension-relative cardiac remodeling. Additionally, in the present study, we observed the significant increase in HIF-1α mRNA relative expression and the decrease in PPARα and CPT-1 mRNA relative expression. The above results suggested that SNS overactivity due to VMH activation might affect the HIF-1α/PPARα/CPT-1 pathway, causing the deterioration of cardiac remodeling.

### Clinical Implication

Evidence supports that the environmental threats causing emotional stress could activate VMH firing (1, 21). Additionally, VMH activation through optogenetics or pharmacogenetics methods could increase anxiety, fear, defensive or aggressive emotional reactions, and VMH inhibition could decrease above emotional reactions (2–4). The above results indicate that the VMH plays the key role in responding to emotional stress. Emotional stresses such as anxiety, fear and anger were the independent risk factors and predictors of hypertension (36). Clinical studies have shown that people under emotional stress would more likely to suffer from hypertension (37, 38). Furthermore, prolonged exposure to emotional stressors would cause poor prognosis of hypertension. Compared with other hypertensive patients, hypertensive patients with higher emotional stress showed blood pressure elevation (39), poor blood pressure control (40), sympathetic nervous system overactivation and cardiac remodeling deterioration (41, 42).

Therefore, evaluating the activity of the VMH through fMRI or PET-CT might help to predict hypertensive patient prognosis. On the other hand, the present study results suggest that pharmacological inhibition of HIF-1α might improve the outcomes of hypertensive patients with emotional stress. This study might provide a novel target for preventing ventricular remodeling in hypertensive patients with emotional stress.

### Limitations

There are several limitations of this research. First, we activated all neurons in the VMH. Whereas, there are multiple kinds of neurons in the VMH. Further research needs to identify the specific role of each kind of neuron in cardiovascular disease. Second, VMH neurons show sexual dimorphisms and could regulate sex-dependent emotional reactions (43, 44). In this study, we demonstrated the effects of VMH activation in male rats, therefore, further study should focus on the cardiovascular effect of VMH activation in the female subjects. Third, we only investigate the effects of VMH activation on early-stage cardiac structure remodeling. In the future research, we will take a longer observation for the cardiac structure and function changes.

## Conclusion

In conclusion, activation of VMH could enhance SBP and aggravate hypertensive relative cardiac remodeling via sympathetic nerve activation, and the underlying mechanism might be the HIF-1α/PPARα/CPT-1 pathway.

## Data Availability Statement

The datasets presented in this study can be found in online repositories. The names of the repository/repositories and accession number(s) can be found at: SRA, PRJNA748709.

## Ethics Statement

The animal study was reviewed and approved by Animal Care and Use Committees of Renmin Hospital of Wuhan University.

## Author Contributions

HJ and XZ designed the study. YZ, ZhL, and ZiL analyzed data and wrote and revised the manuscript. HZ, XX, ZeL, and HC performed the animal experiments and statistical analysis. YW, ZZ, MW, YL, and LZ edited manuscript. All authors contributed to the article and approved the submitted version.

## Funding

This work was supported by the National Key R&D Program of China (2017YFC1307800) and the National Natural Science Foundation of China (81970287, 81530011, and 81770364).

## Conflict of Interest

The authors declare that the research was conducted in the absence of any commercial or financial relationships that could be construed as a potential conflict of interest.

## Publisher's Note

All claims expressed in this article are solely those of the authors and do not necessarily represent those of their affiliated organizations, or those of the publisher, the editors and the reviewers. Any product that may be evaluated in this article, or claim that may be made by its manufacturer, is not guaranteed or endorsed by the publisher.

## Abbreviations

VMH: Ventromedial hypothalamus
CVDs: Cardiovascular diseases
SNS: Sympathetic nervous system
DREADDs: Designer receptors exclusively activated designer drugs
HTN: Hypertension
2K1C, Two-kidney: one-clip
CNO: Clozapine-N-oxide
3V: The third ventricle
SBP: Systolic blood pressure
ANP: Atrial natriuretic peptide
BNP: Brain natriuretic peptide
HRV: Heart rate variability
HF: High frequency
LF: Low frequency
LF/HF: The ratio between LF and HF
NE: Norepinephrine
HIF-1α: Hypoxia-inducible factor-1α
PPARα: Peroxisome proliferator-activated receptorα
CPT-1: Carnitine palmitoyl transferase 1
GO: Gene ontology
KEGG: Kyoto encyclopedia of genes and genomes.

## References

[1] Mendes-Gomes J Motta SC Passoni Bindi R de Oliveira AR Ullah F Baldo MVC. Defensive behaviors and brain regional activation changes in rats confronting a snake. Behav Brain Res. (2020) 381:112469. 10.1016/j.bbr.2020.112469 31917239

[2] Viskaitis P Irvine EE Smith MA Choudhury AI Alvarez-Curto E Glegola JA. Modulation of SF1 neuron activity coordinately regulates both feeding behavior and associated emotional states. Cell Rep. (2017) 21:3559–72. 10.1016/j.celrep.2017.11.089 29262334 PMC5746599

[3] Falkner AL Grosenick L Davidson TJ Deisseroth K Lin D. Hypothalamic control of male aggression-seeking behavior. Nat Neurosci. (2016) 19:596–604. 10.1038/nn.4264 26950005 PMC4853470

[4] Wang L Chen IZ Lin D. Collateral pathways from the ventromedial hypothalamus mediate defensive behaviors. Neuron. (2015) 85:1344–58. 10.1016/j.neuron.2014.12.025 25754823 PMC4368499

[5] Kivimäki M Steptoe A. Effects of stress on the development and progression of cardiovascular disease. Nat Rev Cardiol. (2018) 15:215–29. 10.1038/nrcardio.2017.189 29213140

[6] Mostofsky E Penner EA Mittleman MA. Outbursts of anger as a trigger of acute cardiovascular events: a systematic review and meta-analysis. Eur Heart J. (2014) 35:1404–10. 10.1093/eurheartj/ehu033 24591550 PMC4043318

[7] Janszky I Ahnve S Lundberg I Hemmingsson T. Early-onset depression, anxiety, and risk of subsequent coronary heart disease: 37-year follow-up of 49,321 young Swedish men. J Am Coll Cardiol. (2010) 56:31–7. 10.1016/j.jacc.2010.03.033 20620714

[8] Moser DK McKinley S Riegel B Doering LV Meischke H Pelter M. Relationship of persistent symptoms of anxiety to morbidity and mortality outcomes in patients with coronary heart disease. Psychosom Med. (2011) 73:803–9. 10.1097/PSY.0b013e3182364992 22021458

[9] Roest AM Martens EJ Denollet J de Jonge P. Prognostic association of anxiety post myocardial infarction with mortality and new cardiac events: a meta-analysis. Psychosom Med. (2010) 72:563–9. 10.1097/PSY.0b013e3181dbff97 20410247

[10] Valensi P Doaré L Perret G Germack R Pariès J Mesangeau D. Cardiovascular vagosympathetic activity in rats with ventromedial hypothalamic obesity. Obes Res. (2003) 11:54–64. 10.1038/oby.2003.10 12529486

[11] Gauthier P Reis DJ Nathan MA. Arterial hypertension elicited either by lesions or by electrical stimulations of the rostral hypothalamus in the rat. Brain Res. (1981) 211:91–105. 10.1016/0006-8993(81)90069-X 7225845

[12] King BM. The rise, fall, and resurrection of the ventromedial hypothalamus in the regulation of feeding behavior and body weight. Physiol Behav. (2006) 87:221–44. 10.1016/j.physbeh.2005.10.007 16412483

[13] Triposkiadis F Karayannis G Giamouzis G Skoularigis J Louridas G Butler J. The sympathetic nervous system in heart failure physiology, pathophysiology, and clinical implications. J Am Coll Cardiol. (2009) 54:1747–62. 10.1016/j.jacc.2009.05.015 19874988

[14] Florea VG Cohn JN. The autonomic nervous system and heart failure. Circ Res. (2014) 114:1815–26. 10.1161/CIRCRESAHA.114.302589 24855204

[15] Mancia G Grassi G Giannattasio C Seravalle G. Sympathetic activation in the pathogenesis of hypertension and progression of organ damage. Hypertension (Dallas, Tex: 1979). (1999) 34:724–8. 10.1161/01.HYP.34.4.724 10523349

[16] Krishnamurthy P Subramanian V Singh M Singh K. Beta1 integrins modulate beta-adrenergic receptor-stimulated cardiac myocyte apoptosis and myocardial remodeling. Hypertension (Dallas, Tex: 1979). (2007) 49:865–72. 10.1161/01.HYP.0000258703.36986.13 17283249

[17] Adamcova M Baka T Dolezelova E Aziriova S Krajcirovicova K Karesova I. Relations between markers of cardiac remodelling and left ventricular collagen in an isoproterenol-induced heart damage model. J Physiol Pharmacol Off J Pol Physiol Soc. (2019) 70:71–7. 10.26402/jpp.2019.1.08 31019126

[18] Yu L Zhou L Cao G Po SS Huang B Zhou X. Optogenetic modulation of cardiac sympathetic nerve activity to prevent ventricular arrhythmias. J Am Coll Cardiol. (2017) 70:2778–90. 10.1016/j.jacc.2017.09.1107 29191327

[19] Chen M Zhou X Yu L Liu Q Sheng X Wang Z. Low-level vagus nerve stimulation attenuates myocardial ischemic reperfusion injury by antioxidative stress and antiapoptosis reactions in canines. J Cardiovas Electrophysiol. (2016) 27:224–31. 10.1111/jce.12850 26546374

[20] Agrawal R Durupt G Verma D Montgomery M Vieira-de Abreu A Taylor C. MicroRNA-7a overexpression in VMH restores the sympathoadrenal response to hypoglycemia. JCI Insight. (2019) 4:e130521. 10.1172/jci.insight.130521 31619588 PMC6824313

[21] Noronha SSR Lima PM Campos GSV Chírico MTT Abreu AR Figueiredo AB. Association of high-fat diet with neuroinflammation, anxiety-like defensive behavioral responses, and altered thermoregulatory responses in male rats. Brain Behav Immun. (2019) 80:500–11. 10.1016/j.bbi.2019.04.030 31022457

[22] Satoh N Ogawa Y Katsuura G Numata Y Tsuji T Hayase M. Sympathetic activation of leptin via the ventromedial hypothalamus: leptin-induced increase in catecholamine secretion. Diabetes. (1999) 48:1787–93. 10.2337/diabetes.48.9.1787 10480609

[23] Sakaguchi T Bray GA. The effect of intrahypothalamic injections of glucose on sympathetic efferent firing rate. Brain Res Bull. (1987) 18:591–5. 10.1016/0361-9230(87)90128-6 3607527

[24] Lim K Barzel B Burke SL Armitage JA Head GA. Origin of aberrant blood pressure and sympathetic regulation in diet-induced obesity. Hypertension (Dallas, Tex: 1979). (2016) 68:491–500. 10.1161/HYPERTENSIONAHA.116.07461 27296999

[25] Takenaka K Sasaki S Uchida A Fujita H Nakamura K Ichida T. GABAB-ergic stimulation in hypothalamic pressor area induces larger sympathetic and cardiovascular depression in spontaneously hypertensive rats. Am J Hyper. (1996) 9:964–72. 10.1016/0895-7061(96)00171-9 8896648

[26] Soñanez-Organis JG Godoy-Lugo JA Hernández-Palomares ML Rodríguez-Martínez D Rosas-Rodríguez JA González-Ochoa G. HIF-1α and PPARγ during physiological cardiac hypertrophy induced by pregnancy: transcriptional activities and effects on target genes. Gene. (2016) 591:376–81. 10.1016/j.gene.2016.06.025 27312951

[27] Krishnan J Suter M Windak R Krebs T Felley A Montessuit C. Activation of a HIF1alpha-PPARgamma axis underlies the integration of glycolytic and lipid anabolic pathways in pathologic cardiac hypertrophy. Cell Metab. (2009) 9:512–24. 10.1016/j.cmet.2009.05.005 19490906

[28] Zhu ZY Wang F Jia CH Xie ML. Apigenin-induced HIF-1α inhibitory effect improves abnormal glucolipid metabolism in AngII/hypoxia-stimulated or HIF-1α-overexpressed H9c2 cells. Phytomed Int J Phytother Phytopharmacol. (2019) 62:152713. 10.1016/j.phymed.2018.10.010 31078968

[29] Niu G Zhou M Wang F Yang J Huang J Zhu Z. Marein ameliorates Ang II/hypoxia-induced abnormal glucolipid metabolism by modulating the HIF-1α/PPARα/γ pathway in H9c2 cells. Drug Dev Res. (2020) 82:523–32. 10.1002/ddr.21770 33314222

[30] Gao X Zhang Z Li X Wei Q Li H Li C. Ursolic acid improves monocrotaline-induced right ventricular remodeling by regulating metabolism. J Cardiovasc Pharmacol. (2020) 75:545–55. 10.1097/FJC.0000000000000815 32141989

[31] He L Kim T Long Q Liu J Wang P Zhou Y. Carnitine palmitoyltransferase-1b deficiency aggravates pressure overload-induced cardiac hypertrophy caused by lipotoxicity. Circulation. (2012) 126:1705–16. 10.1161/CIRCULATIONAHA.111.075978 22932257 PMC3484985

[32] Huang Z Li G Zhang Z Gu R Wang W Lai X. β2AR-HIF-1α-CXCL12 signaling of osteoblasts activated by isoproterenol promotes migration and invasion of prostate cancer cells. BMC Cancer. (2019) 19:1142. 10.1186/s12885-019-6301-1 31771535 PMC6878637

[33] Koeners MP Vink EE Kuijper A Gadellaa N Rosenberger C Mathia S. Stabilization of hypoxia inducible factor-1α ameliorates acute renal neurogenic hypertension. J Hyper. (2014) 32:587–97. 10.1097/HJH.0000000000000060 24309492

[34] Barger PM Brandt JM Leone TC Weinheimer CJ Kelly DP. Deactivation of peroxisome proliferator-activated receptor-alpha during cardiac hypertrophic growth. J Clin Investig. (2000) 105:1723–30. 10.1172/JCI9056 10862787 PMC378509

[35] Young ME Laws FA Goodwin GW Taegtmeyer H. Reactivation of peroxisome proliferator-activated receptor alpha is associated with contractile dysfunction in hypertrophied rat heart. J Biol Chem. (2001) 276:44390–5. 10.1074/jbc.M103826200 11574533

[36] Rutledge T Hogan BE. A quantitative review of prospective evidence linking psychological factors with hypertension development. Psychosom Med. (2002) 64:758–66. 10.1097/01.PSY.0000031578.42041.1C 12271106

[37] Player MS King DE Mainous AG 3rd Geesey ME. Psychosocial factors and progression from prehypertension to hypertension or coronary heart disease. Ann Fam Med. (2007) 5:403–11. 10.1370/afm.738 17893381 PMC2000300

[38] Sumner JA Kubzansky LD Roberts AL Chen Q Rimm EB Koenen KC. Not all posttraumatic stress disorder symptoms are equal: fear, dysphoria, and risk of developing hypertension in trauma-exposed women. Psychol Med. (2020) 50:38–47. 10.1017/S0033291718003914 30606272 PMC6609506

[39] Myburgh CE Malan L Wentzel A Scheepers JW Malan NT. Coping and cardiac troponin t–a risk for hypertension and sub-clinical ECG left ventricular hypertrophy: the SABPA study. Heart Lung Circ. (2019) 28:908–16. 10.1016/j.hlc.2018.05.101 29895484

[40] Sanz J García-Vera MP Espinosa R Fortún M Magán I Segura J. Psychological factors associated with poor hypertension control: differences in personality and stress between patients with controlled and uncontrolled hypertension. Psychol Rep. (2010) 107:923–38. 10.2466/09.15.20.PR0.107.6.923-938 21323151

[41] Kozul K Vidović K Heinzelman-Kozul H Samardzić S Kopić M Sram M. The heart frequency and its variability in hypertensive patients considering A/B type of behaviour and eight basic emotions and levels of anger expression. Coll Antropol. (2009) 33:409–16. 19662757

[42] Munakata M Hiraizumi T Nunokawa T Ito N Taguchi F Yamauchi Y. Type A behavior is associated with an increased risk of left ventricular hypertrophy in male patients with essential hypertension. J Hyper. (1999) 17:115–20. 10.1097/00004872-199917010-00017 10100102

[43] Cheung CC Krause WC Edwards RH Yang CF Shah NM Hnasko TS. Sex-dependent changes in metabolism and behavior, as well as reduced anxiety after eliminating ventromedial hypothalamus excitatory output. Mol Metab. (2015) 4:857–66. 10.1016/j.molmet.2015.09.001 26629409 PMC4632173

[44] Yang CF Chiang MC Gray DC Prabhakaran M Alvarado M Juntti SA. Sexually dimorphic neurons in the ventromedial hypothalamus govern mating in both sexes and aggression in males. Cell. (2013) 153:896–909. 10.1016/j.cell.2013.04.017 23663785 PMC3767768

