# Supplementary material for: Ventromedial Hypothalamus Activation Aggravates Hypertension Myocardial Remodeling Through the Sympathetic Nervous System
Source: Front Cardiovasc Med. 2021 Oct 18;8:737135. doi: 10.3389/fcvm.2021.737135 (PMC8558385; doi:10.3389/fcvm.2021.737135)
Supplement: Supplementary file 1 [file Data_Sheet_1.docx]

**Supplemental Methods**

At 5 w, transthoracic ultrasonic cardiogram was performed under anesthesia with 2.5% [pentobarbital](javascript:;) by using High Resolution Imaging System machine (GE Vivid E95, USA) equipped with a 12-MHz probe (12S). Standard M-mode images were taken in the long- and short-axis positions at mitral valve tip level under the guidance of two-dimensional images. Left ventricular end-systolic diameter (LVIDs), left ventricular end-diastole diameter (LVIDd), ejection fraction (EF) and fractional shortening (FS) were measured.

**Supplemental Results and Figures**

Compared with the sham group, there were significantly decreases of EF and FS in the HTN group (EF, 94.01±0.99% vs. 89.37±0.50%, sham vs. HTN, *p*<0.05; FS, 62.91±2.19% vs. 54.15±0.72%, sham vs. HTN, *p*<0.05). Moreover, the decreases in EF and FS were statistically aggravated by VMH activation (EF, 89.37±0.50% vs. 81.17±1.67%, HTN vs. HTN+VMH activation, *p*<0.01; FS, 54.15±0.72% vs. 44.88±1.70%, HTN vs. HTN+VMH activation, *p*<0.01, Figure S1-C and S1-D). Compared with the HTN group, the HTN+VMH activation group also showed increased LVIDs (LVIDs, 2.18±0.06mm vs. 3.10±0.24mm, HTN vs. HTN+VMH activation, *p*<0.05, Figure S1-A). There were no significantly changes in LVIDs between the sham group and the HTN group (*p*>0.05, Figure S1-A). No statistical changes were observed in LVIDd in all three group (*p*>0.05, Figure S1-B).


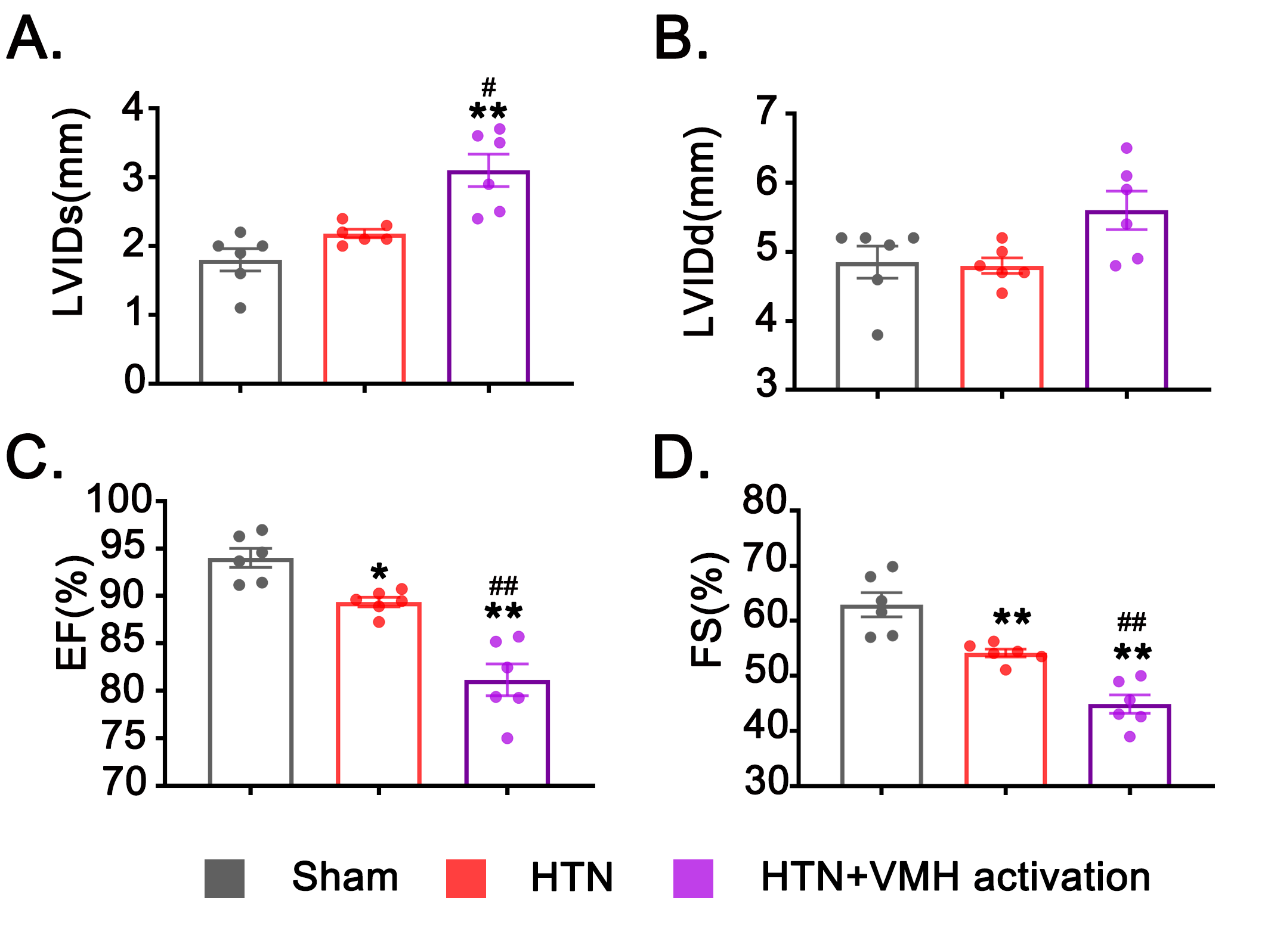


**Supplementary Figure S1** VMH activation increased LVIDs and decreased EF as well as FS. **A-D.** Echocardiograph results of LVIDs, LVIDd, EF and FS were detected at 5W. Values are presented as mean ± SEM (n=6 per group). LVIDs, left ventricular end-systolic diameter; LVIDd, left ventricular end-diastole diameter; EF, left ventricular ejection fraction; FS, fractional shortening. **p*<0.05, ** *p* <0.01 vs. sham group; # *p* <0.05, # # *p* <0.01 vs. HTN group.
